# Deaths due to malnutrition in children under five in Colombia, 2015–2023: a spatiotemporal analysis

**DOI:** 10.3389/fpubh.2026.1816399

**Published:** 2026-06-22

**Authors:** Deissy R. Agudelo-Ibáñez, Daniel Diaz, Maylen L. Rojas-Botero, Julián A. Fernández-Niño, Javier H. Eslava-Schmalbach, Lina S. Palacio-Mejía

**Affiliations:** 1 Escuela de Salud Pública de México, Instituto Nacional de Salud Pública, Cuernavaca, Morelos, Mexico; 2 Centro de Investigación en Evaluación y Encuestas, Instituto Nacional de Salud Pública, Cuernavaca, Morelos, Mexico; 3 Facultad Nacional de Salud Pública, Universidad de Antioquia, Medellín, Colombia; 4 Secretaría Distrital de Salud, Bogotá, Colombia; 5 Departamento de Salud Pública, Universidad del Norte, Barranquilla, Colombia; 6 Facultad de Medicina, Universidad Nacional de Colombia, Sede Bogotá, Colombia

**Keywords:** child malnutrition, health trends, infant mortality, regional disparities, spatial analysis

## Abstract

**Introduction:**

In Colombia, child mortality due to malnutrition persists as a public health problem that has not been comprehensively analyzed over the past decade.

**Objective:**

To provide a comprehensive analysis of the spatiotemporal behavior of mortality due to malnutrition in children under 5 years of age (CU5) in Colombia at the national and subnational levels disaggregated by sex and age group from 2015 to 2023.

**Methodology:**

An ecological study was conducted using secondary sources. Mortality due to malnutrition was defined as any death in CU5 whose underlying cause was coded as D50-D53, E40-E64, or P07 of the ICD-10. The study period begins in 2015 to ensure data uniformity and provide a stable baseline following the implementation of the 2018 National Population and Housing Census projections. The mortality rates were calculated in CU5, children under 1 year of age (CU1), children aged 1–4 years of age (C1-4) per 100,000 children. A descriptive analysis was performed, maps were created, and temporal trends were identified using joinpoint regression.

**Results:**

From 2015 to 2023, there were 75,068 deaths from all causes in CU5, of which 2,694 (3.6%) were due to malnutrition. The most affected age group was CU1 (57.8–70.2% of annual malnutrition deaths), with males accounting for 54.1% of deaths. The national mortality rate due to malnutrition in CU5 fluctuated between 3.1 and 5.0 deaths per 100,000 children, with the highest value occurring in 2022. Notable increases in mortality rates were observed in La Guajira, Vichada, and Chocó Departments, with the latter rising from the 4th to the 1st national rank (reaching 77.9 deaths per 100,000 children in 2023), indicating marked geographical disparities.

**Conclusion:**

Deaths from malnutrition in CU5 in Colombia demonstrated disparities by sex, age, and geographical location, with heterogeneous trends observed over the period. A comprehensive understanding of these disparities can inform targeted interventions aimed at reducing child mortality from malnutrition in the country.

## 1 Introduction

Malnutrition in children under 5 years of age (CU5), remains one of the main public health problems worldwide. According to the World Health Organization (WHO) and the Food and Agriculture Organization of the United Nations (FAO), in 2024 approximately 150 million CU5 were stunted (chronic malnutrition), while nearly 43 million were wasted (1). In affected individuals, malnutrition causes impaired cognitive and physical development, contributing to lost productivity in adulthood, thus perpetuating cycles of poverty and inequality (2). In addition, stunting, wasting, and underweight during early childhood increase the risk of death by up to 12 times (3), accounting for approximately 53% of deaths in CU5 (4). In 2021, it was estimated that the number of deaths globally attributable to malnutrition in CU5 reached up to 774,000, representing 16.6% of all deaths recorded in this age group (5).

In particular, children under 1 year of age (CU1) are the most affected by deaths associated with malnutrition, whose basic and underlying causes at the individual level include short gestation, low birth weight, protein-calorie malnutrition, and recurrent infections (6). At the structural level, low family income, limited education of caregivers, difficulties in accessing health services, and inequalities related to ethnicity and gender are among the main social determinants that strengthen the link between poverty and malnutrition (7–9). In Colombian rural areas, food insecurity has been identified as a key driver of the double burden of malnutrition, which is characterized by the coexistence of stunted children and overweight or obesity in adults, reflecting the complex nutritional transition occurring in the country (10). This situation is prevalent in low- and middle-income countries, where 6.6% of CU5 are emaciated, which is the form of malnutrition most associated with fatal outcomes (1). In Colombia, a middle-income country, despite the nutritional transition (11), malnutrition levels in CU5 remain a persistent public health concern. According to the most recent National Nutritional Status Survey (ENSIN 2015), 1.6% of CU5 suffered from acute malnutrition, 3.7% from global malnutrition, and 10.8% from chronic malnutrition, exceeding figures for other countries in the region such as Chile, Peru, Guatemala, Mexico, and Honduras (12). A recent study confirmed this trend in the Department of Antioquia, where malnutrition was the second leading cause of death among CU5 in 2021, with a mortality rate of 2.2 deaths per 100,000 children, just below acute respiratory insufficiency (13).

Despite the existence of national studies, which describe the behavior of mortality due to malnutrition in CU5 in Colombia (14, 15), to date, no subnational analysis has been conducted, nor has any updated data been released. Furthermore, given that this disease is both preventable and treatable through timely interventions, most deaths due to malnutrition are avoidable. An initial step is to understand the current epidemiology of mortality due to malnutrition in CU5, including its trends and spatial distribution disaggregated by sex and age group. Accordingly, the present study aims to describe the spatiotemporal behavior of mortality due to malnutrition in CU5 in Colombia between 2015 and 2023. This information will be essential for determining the presence of territorial inequalities, identifying vulnerable groups, and assessing the existence of gaps in the strengthening of primary care. The results of the research are expected to provide evidence for public health decision-making, guiding the targeting of interventions such as the promotion of exclusive breastfeeding and nutrition education in priority populations and localities. In addition, the information from this study could be useful for the implementation of active nutritional surveillance programs and the generation of intersectoral public policies on food security (16).

## 2 Methods

### 2.1 Study design and case definition

A multitemporal and multispatial ecological observational study (17) was conducted using secondary sources to describe the behavior of mortality from malnutrition in CU5 in Colombia during the period 2015 to 2023 at the national and departmental levels by sex and age group. For this study, the units of analysis were the 32 departments of Colombia identified according to the political-administrative division codes (DIVIPOLA) provided by the National Administrative Department of Statistics (DANE). The study period begins in 2015 to ensure data uniformity, as this year follows the standardization of vital statistics reporting by DANE and provides a stable baseline prior to the 2018 National Population and Housing Census, from which population projections were derived.

For this study, records of deaths in CU5 from all causes were used, and deaths due to malnutrition were extracted from these records. In Colombia, mortality from malnutrition in CU5 is monitored by the National Institute of Health (INS), which defines it as “*any death of a child under five whose cause or causes of death include malnutrition and/or nutritional deficiencies”* (18). In this regard, in our study, mortality due to malnutrition was defined as any death of CU5 whose underlying cause of death included malnutrition, nutritional deficiencies, and/or nutritional anemia reported on the death certificate in accordance with the International Statistical Classification of Diseases and Related Health Problems (ICD-10) with codes D50-53 and E40-64 (Table 1). In addition, code P07 (disorders related to short gestation and low birth weight) was included, given that low birth weight is an early manifestation of maternal-fetal nutritional deficiency and is part of the continuum of child malnutrition. These disorders represent a key intermediate pathway between maternal malnutrition, intrauterine growth restriction, and increased vulnerability to mortality in the first year of life.

**Table 1:** ICD-10 codes for causes of mortality due to malnutrition.

Code	Description of the disease
D50	Iron deficiency anemia
D52	Folate deficiency anemia
D53	Other nutritional anemias
E40	Kwashiorkor
E41	Nutritional marasmus
E42	Marasmic kwashiorkor
E43	Severe protein-calorie malnutrition, unspecified
E46	Unspecified protein-calorie malnutrition
E63	Other nutritional deficiencies
E64.0 to 0.9	Sequelae of malnutrition and other nutritional deficiencies
P07	Disorders related to short duration of gestation and low birth weight

In this study, mortality due to malnutrition was defined based on the underlying cause of death recorded on death certificates and coded according to ICD-10, using the official mortality registry as the data source. It is important to acknowledge that ICD-10 coded mortality data do not differentiate between acute and chronic malnutrition based on duration or anthropometric indicators such as stunting, wasting, or underweight. The classification relies exclusively on clinical diagnoses documented on death certificates (e.g., kwashiorkor, marasmus, protein-calorie malnutrition), micronutrient deficiencies, and disorders associated with short gestation and low birth weight. Consequently, the various clinical categories of malnutrition were analyzed in aggregate as causes of death from malnutrition in CU5, without distinguishing between acute and chronic forms or between different temporal trajectories of the condition. This means it was not possible to differentiate, for example, chronic malnutrition in a child of 4.5 years of age from an acute condition in a newborn, since the vital statistics system does not capture anthropometric indicators or details on the duration of the clinical course. This aggregate approach follows the case definition established by the Colombian National Institute of Health (INS) for surveillance purposes and has been validated in previous studies in Colombia (19, 20).

### 2.2 Data sources and study population

Data on mortality and births were taken from Vital Statistics (EEVV) available at: https://microdatos.dane.gov.co/index.php/catalog/DEM-Microdatos, which corresponds to the official registry administered by DANE, where the occurrence of specific vital events in Colombia, as well as their characteristics, are reported (21). In the case of deaths, DANE takes death certificates and codes the causes of death and personal information of the deceased. In this study, we selected records of deaths that occurred in CU5 from all causes with reported residence in Colombia on the date of death and analyzed those deaths whose underlying cause of death was classified with one of the codes described in Table 1.

In addition, population projections made by DANE based on the 2018 Colombia National Population and Housing Census (CNPV) were used, which provide annual estimates of the resident population in Colombia disaggregated at the municipal, departmental, and national levels and contain information by area (urban, rural), biological sex (male and female), and age (22). In this study, the definition of deaths due to malnutrition was based on the information recorded on death certificates and on the underlying cause of death coded according to ICD-10, using the official mortality registry as the data source. Consequently, the different clinical categories of malnutrition were analyzed in aggregate as causes of death from malnutrition in CU5, without distinguishing between acute and chronic forms or between different temporal trajectories of the condition. As the information system does not capture anthropometric indicators or details on the duration of the clinical course, it was not possible to differentiate, for example, chronic malnutrition in older children from acute conditions in newborns.

### 2.3 Mortality rates

From the sources of information, the number of deaths in CU5 from all causes was extracted, and within these, deaths in this age group that reported malnutrition as the underlying cause were identified. In addition, mortality rates due to malnutrition were calculated for the following three age groups: (1) *children under 5 years of age* (CU5 per 100,000 children), (2) *children under 1 year of age* (CU1, per 100,000 live births), and (3) *children aged 1–4 years of age* (C1-4 per 100,000 children). To calculate the rates, the number of deaths from malnutrition in each age group was used as the numerator, while the population projections for each group were used as the denominator, multiplying the quotient by 100,000. Estimates were made every 6 months at the national level with the intention of describing the behavior in greater detail, while annual estimates were used for the 32 departments of Colombia; in both cases, the annual population was used as the denominator for the calculation. A breakdown by sex and age group was performed.

### 2.4 Statistical analysis

A descriptive analysis of mortality due to malnutrition in CU5 was performed, including the construction of time series for the number of deaths and rates per 100,000 children, as well as their percentage distribution by sex and age group, including the calculation of the M:F ratio. In addition, to illustrate the changes in the indicators from 2015 to 2023, heat maps were constructed with arrow diagrams and graphs comparing the change in the mortality rate at the departmental level for each age group in the period studied. Furthermore, to compare the spatial distribution of the mortality rate due to malnutrition at the departmental level for each age group (CU5, CU1, and C1-4), the departmental values were distributed according to their quintile into high, medium-high, medium, medium-low, and low. Based on the distribution of the quintile categories, maps were constructed with departmental representation for Colombia in 2015 and 2023. For this purpose, the online tool Datawrapper was used, available at the following page: https://www.datawrapper.de.

To analyze trends in mortality due to malnutrition in CU5, the percentage difference was calculated, taking the 2015 baseline, using the following formula:

Percentage difference = 100*(Value − Baseline)/Baseline.

This analysis allowed to evaluate the temporal evolution of mortality in CU5 because, if there was a decrease over time with respect to the baseline (2015), the result was expressed as a negative percentage difference. Conversely, when mortality increased, a positive percentage difference was obtained.

Additionally, to evaluate apparent changes in trends in mortality rates among CU5 due to malnutrition at the departmental level between 2015 and 2023, regression analyses were performed using joinpoint models. Joinpoint Trend Analysis 5.4 software, available at https://surveillance.cancer.gov/joinpoint/download (Accessed January 10, 2026), was used for this purpose. The software fits the simplest joinpoint model, starting with a straight line (0 joinpoints), and then tests whether the inclusion of more joinpoints is necessary to explain whether a change in the trend is statistically significant. Based on crude annual rates (per 100,000), the model estimates the annual percentage change (APC) with its 95% confidence interval (95% CI) for each regression segment, assuming a constant rate over the period and testing whether the APC differs significantly from zero using a two-tailed *t-test* at a value of *p* = 0.05 (23). APC values were used to assess the direction and magnitude of change in mortality trends in CU5 due to malnutrition at the departmental level only for the groups of CU5 and CU1. In all cases, only time series with a maximum of two missing data points and in non-consecutive years were included. For this reason, C1-4 age group was excluded from the analysis due to the low number of cases reported in 15/32 departments. Likewise, the departments of Guaviare, Quindío, and Vaupés were excluded from the analysis of one or both age groups due to a lack of data for the model.

### 2.5 Ethical considerations

The research protocol for this study was evaluated by the institutional ethics committee of the National Institute of Public Health of Mexico (INSP, 17CEI00420160708), where it was declared exempt from review (17CEI00420160708). The study was based on secondary information from records anonymized by DANE prior to access for research purposes. Compliance with current national and international ethical standards for health research was always ensured.

## 3 Results

### 3.1 All-cause mortality and deaths due to malnutrition in CU5 in Colombia from 2015 to 2023

**Figure 1:**
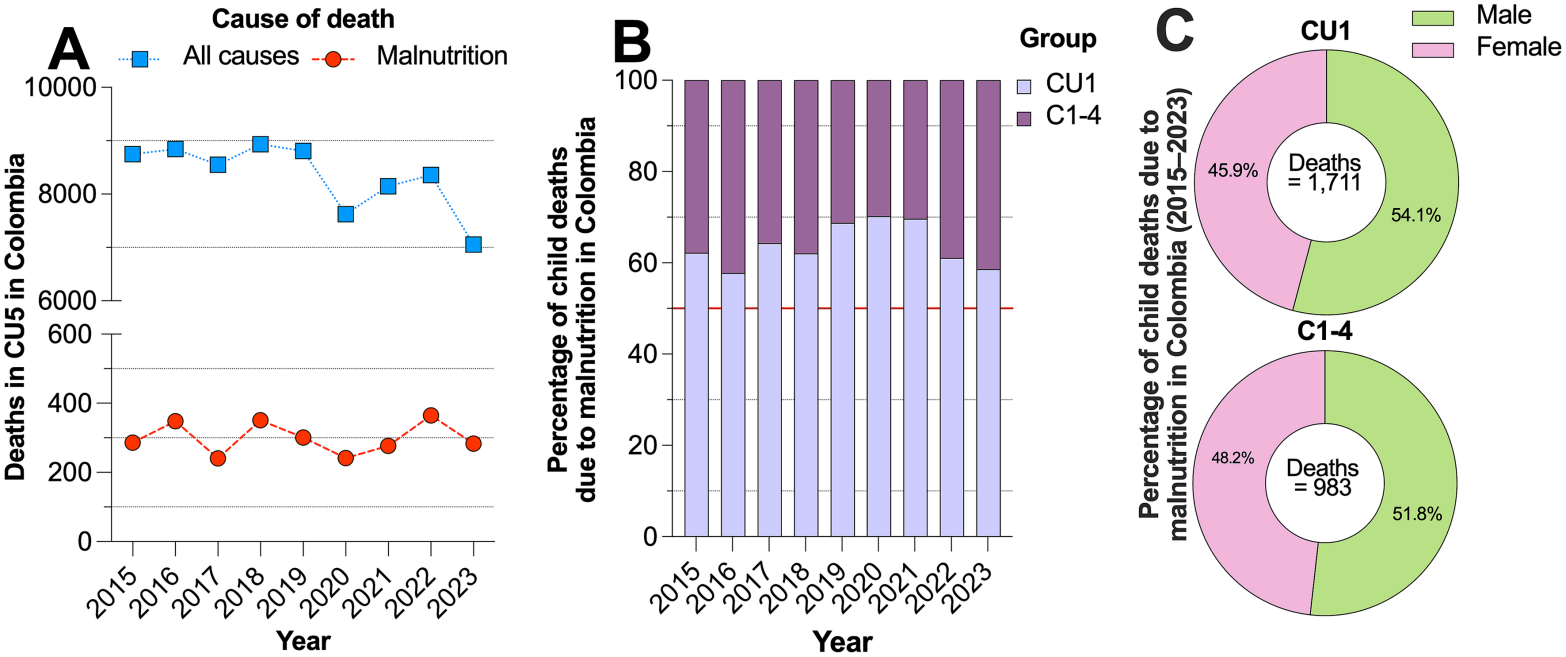
All-cause mortality and deaths due to malnutrition-related mortality in CU5 in Colombia, 2015–2023. **(A)** Temporal trend in CU5 mortality, **(B)** relative contribution of mortality due to malnutrition by age group, and **(C)** distribution of deaths due to malnutrition according to sex and age group.

In Colombia, the total number of deaths from all causes in the general population (men and women of all ages) was estimated at 2,371,065 from 2015 to 2023. During this period, 75,068 CU5 died from all causes, of which 3.6% of deaths (2,694) were directly attributed to malnutrition (Table 2). At the national level, the temporal trend in CU5 mortality varied depending on the underlying cause of death; while all-cause mortality showed a downward trend, deaths due to malnutrition exhibited a heterogeneous behavior (Figure 1A). In 2015, malnutrition was the cause of 286 deaths in CU5, accounting for 3.3% of the total 8,746 deaths recorded among this age group during that year and by 2023, this percentage had increased to 4.0% (283 out of a total of 7,056 deaths). The analysis of the percentage difference, taking 2015 as the baseline, indicated that all-cause mortality in CU5 remained stable at the national level until 2019 and then experienced a variable reduction from 2020 to 2022, reaching a maximum decrease of 19.3% in 2023. Conversely, deaths due to malnutrition exhibited a contrasting trend, with increases exceeding 20% in 2016, 2018, and 2022 (range, 21.6–27.7%) concomitant with declines ranging from 1.1 to 15.7% in the intervening years.

**Table 2:** All-cause mortality and deaths due to malnutrition by age group in Colombia from 2015 to 2023.

Year	All-cause mortality and deaths due to malnutrition in CU5			Deaths due to malnutrition in CU1			Deaths due to malnutrition in C1-4		
	All-cause mortality	Deaths due to malnutrition	CU5MR per 100,000	*n*	CU1MR per 100,000	M:F ratio	*n*	C1-4MR per 100,000	M:F ratio
2015	8,746	286	7.62	178	26.92	1.54	108	3.59	1.25
2016	8,845	348	9.27	201	31.04	0.91	147	4.89	2.25
2017	8,548	241	6.41	155	23.60	0.93	86	2.85	1.15
2018	8,933	351	9.21	218	33.58	0.98	133	4.36	0.90
2019	8,809	301	7.75	207	32.21	1.35	94	3.02	1.29
2020	7,624	242	6.28	170	27.01	1.36	72	2.32	1.40
2021	8,147	277	7.24	193	31.28	1.05	84	2.71	0.65
2022	8,360	365	9.69	223	38.87	1.16	142	4.65	1.12
2023	7,056	283	7.64	166	32.19	1.63	117	3.91	1.21
Total	75,068	2,694		1,711			983		

CU5, children under five; CU5MR, children under five mortality rate due to malnutrition; CU1, children under 1; CU1MR, children under 1 mortality rate due to malnutrition; C1-4, children aged 1–4; C1-4MR, children aged 1–4 mortality rate due to malnutrition; M:F, male-to-female ratio of deaths due to malnutrition.

CU1 consistently accounted for the highest burden of mortality due to malnutrition in comparison with C1-4, representing between 57.8 and 70.2% of deaths across the period evaluated (Figure 1B). Furthermore, mortality was slightly higher for males than for females in both age groups (Figure 1C) because of the 1,711 deaths in CU1, 54.1% (926) occurred among males, and 51.8% (509) of the deaths recorded in the C1-4 group occurred among males. Such a trend possibly reflects the national distribution of births by sex, which according to the DANE, remained constant from 2018 to 2022 (24). Of the total 569,311 births recorded in 2022, 51.1% were males and 48.9% females (24).

In CU1, the male-to-female ratio (M:F) showed variability over the studied period (Table 2). In 2015, the ratio was 1.54, indicating higher mortality among males, with the ratio falling below 1.0 from 2016 to 2018, indicating excess female mortality during those years. Beginning in 2019, the M:F ratio exhibited a consistent upward trend, remaining above 1.0 until its peak in 2023 (1.63). In the C1-4 group, M:F ratio also exhibited variability, with values ranging from 0.65 (in 2021, indicating excess female mortality) to 2.25 (in 2016, indicating marked excess male mortality). Therefore, no consistent trend was identified in either group, indicating that vulnerability by sex exhibits variability over time and varies by age.

Between 2015 and 2023, Colombia exhibited a consistent decline in both the mortality rate (per 100,000 children) of CU5 and CU1. The former decreased from 15.5 deaths in 2015 to 12.0 in 2023, representing a relative reduction of 22.6% over the study period, whereas the latter followed a parallel trajectory, declining from 13.9 to 10.8 deaths, equivalent to a relative reduction of 22.3%.

**Figure 2:**
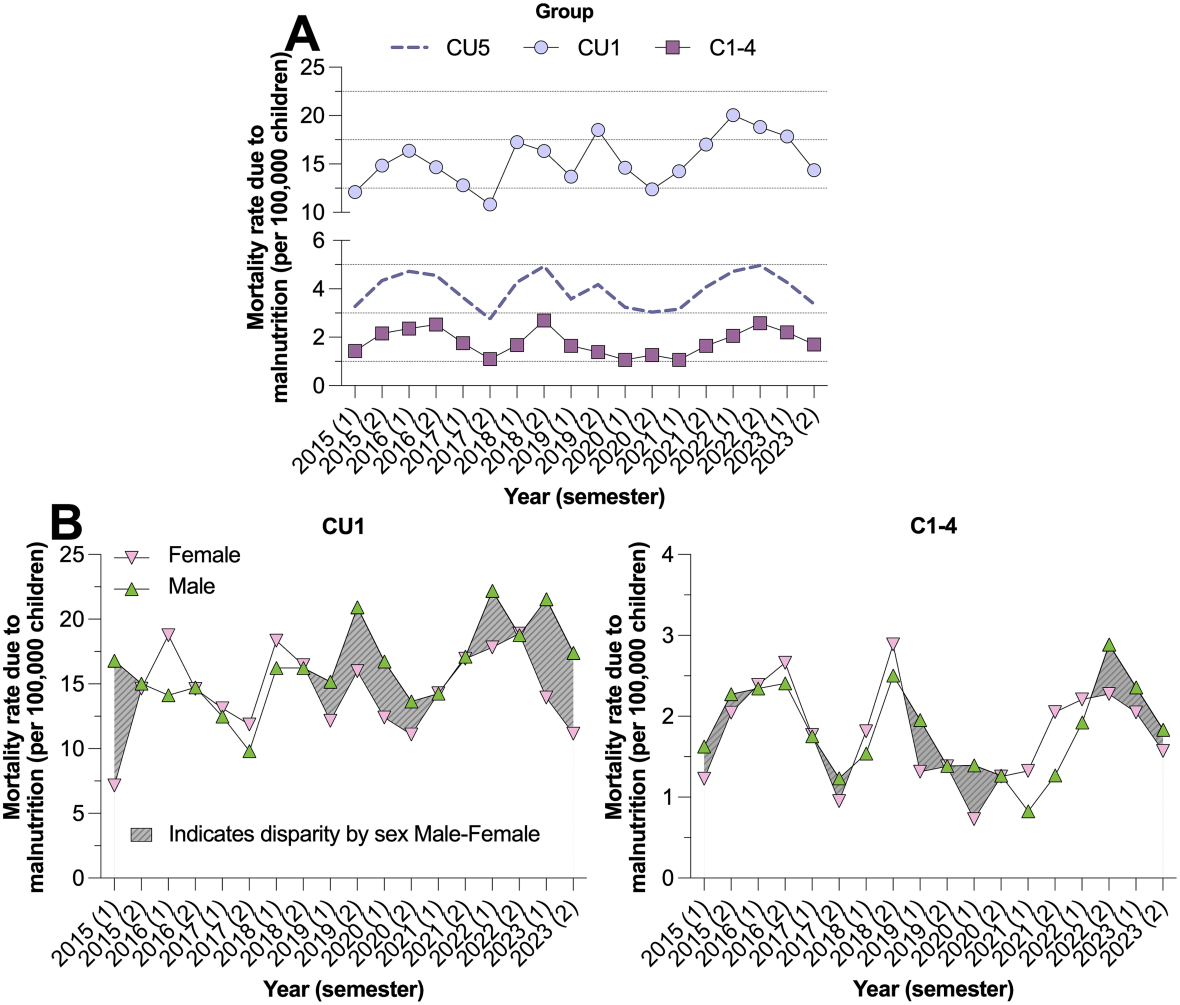
Evolution of the mortality rate due to malnutrition in CU5 in Colombia, 2015–2023. **(A)** Trend in the mortality rate (per 100,000 children) due to malnutrition per semester by age group, and **(B)** time series of the mortality rate due to malnutrition (per 100,000 children) by sex and age group.

At the national level, the mortality rate due to malnutrition (per 100,000 children) exhibited a fluctuating and highly heterogeneous pattern from 2015 to 2023, with substantial differences in the estimates between age groups across the years (Figure 2A). The rate was higher in CU1 compared to the C1-4 group, confirming that the highest burden of this outcome was concentrated in the first year of life. Among CU1, an increase was observed during the latter half of 2022, with a rate of 5.0 deaths per 100,000 children. A similar trend was noted in CU1, though on a higher magnitude (20.1 deaths per 100,000). Throughout the period, the mortality rate due to malnutrition among C1-4 was consistently the lowest, ranging from 1.1 to 2.7 deaths per 100,000 children. A subsequent analysis by sex and age group revealed inter-semester variations without a discernible pattern of increase or decrease over the period 2015–2023 (Figure 2B). While the mortality rates due to malnutrition exhibited fluctuations between males and females in both age groups, males in the CU1 group had a tendency to exhibit higher values and more pronounced peaks in comparison to females, as evidenced in the initial half of 2022 (girls 17.8 vs. boys 22.2) and 2023 (girls 13.9 vs. boys 21.5; Figure 2B, left panel). Conversely, in C1-4, there was no sustained gap by sex, but rather an alternation in the greater vulnerability between sexes over time (Figure 2B, right panel).

### 3.2 Spatial distribution of the mortality rate due to malnutrition in children aged 0–4 years by department in Colombia from 2015 to 2023

**Figure 3:**
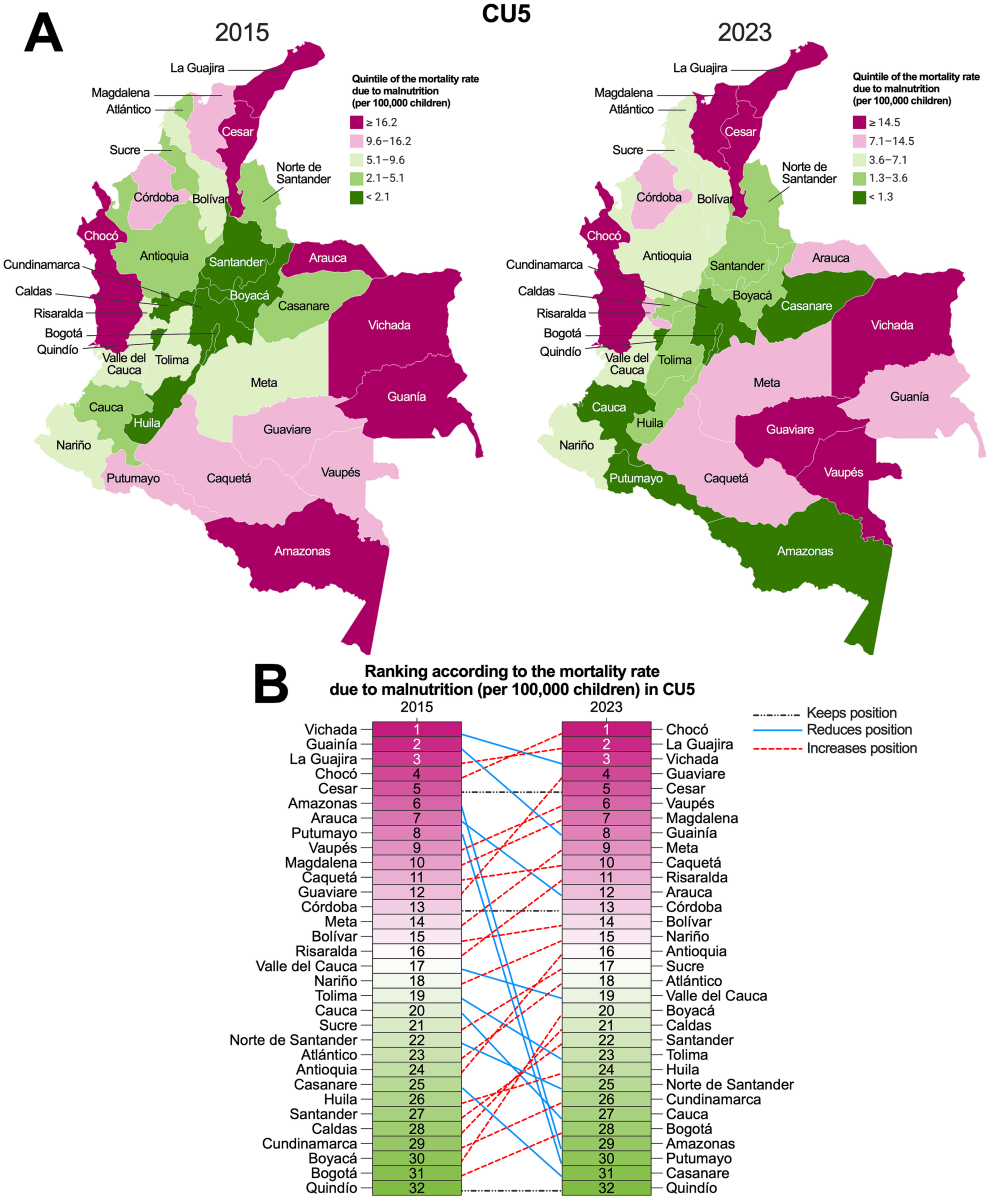
Temporal change in the mortality rate due to malnutrition in CU5 by department in Colombia between 2015 and 2023. **(A)** Spatial distribution of the quintile of the mortality rate (per 100,000 children) due to malnutrition in 2015 and 2023 and **(B)** relative change in the position of each department according to the mortality rate (per 100,000 children) in CU5 in 2015 and 2023.

Figure 3A illustrates the spatial distribution of the mortality rate (per 100,000 children) due to malnutrition in CU5 in Colombia in 2015 and 2023. At the beginning of the period, the rate was low in the center and west of the country and medium-high in the peripheries (Figure 3A, left panel). The departments of Vichada, Guainía, Arauca, Cesar, and La Guajira, as well as Chocó and Amazonas, were in the highest quintile, with ≥16.2 deaths per 100,000 children. In the southern region, a cluster was identified in the Colombian Amazon comprising the departments of Vaupés, Caquetá, Guaviare, and Putumayo. These departments exhibited mortality rates that placed them in the second highest quintile, with rates ranging from 9.6 to 16.2 deaths per 100,000 children. In contrast, the cluster comprising Boyacá, Santander, Bogotá, Cundinamarca, Caldas, and Quindío, located in the central region of the country was included in the lowest quintile, with a mortality rate of less than 2.1 deaths. Despite an overall decline in mortality rates during 2023, territorial inequalities persisted (Figure 3A, right panel). The departments of La Guajira, Cesar, Vichada, and Chocó remained in the top of the quintile (≥14.5 deaths), while Magdalena, Vaupés, and Guaviare ascended to this group, thereby establishing novel hotspots of elevated mortality due to malnutrition in CU5. At the center of the country, the departments of Bogotá, Quindío, and Cundinamarca persisted in the lowest quintile (<1.3 deaths per 100,000), confirming the regional disparities.

Figure 3B summarizes the relative position of the departments regarding their mortality rates due to malnutrition in CU5 in 2015 and 2023. Ten of the departments experienced a decline in their ranking, three maintained their position, and 19 ascended in the rankings. Although Vichada, La Guajira, Chocó, and Cesar maintained their positions within the top five departments with the highest rates, Amazonas and Putumayo exhibited a substantial decrease in their positions, dropping from the top 10 to 29th and 30th place, respectively. The three departments that showed substantial increases were Guaviare and Antioquia, which ascended eight positions each, meanwhile Boyacá ascended from the 30th position to the 20th, thus climbing 10 places.

**Figure 4:**
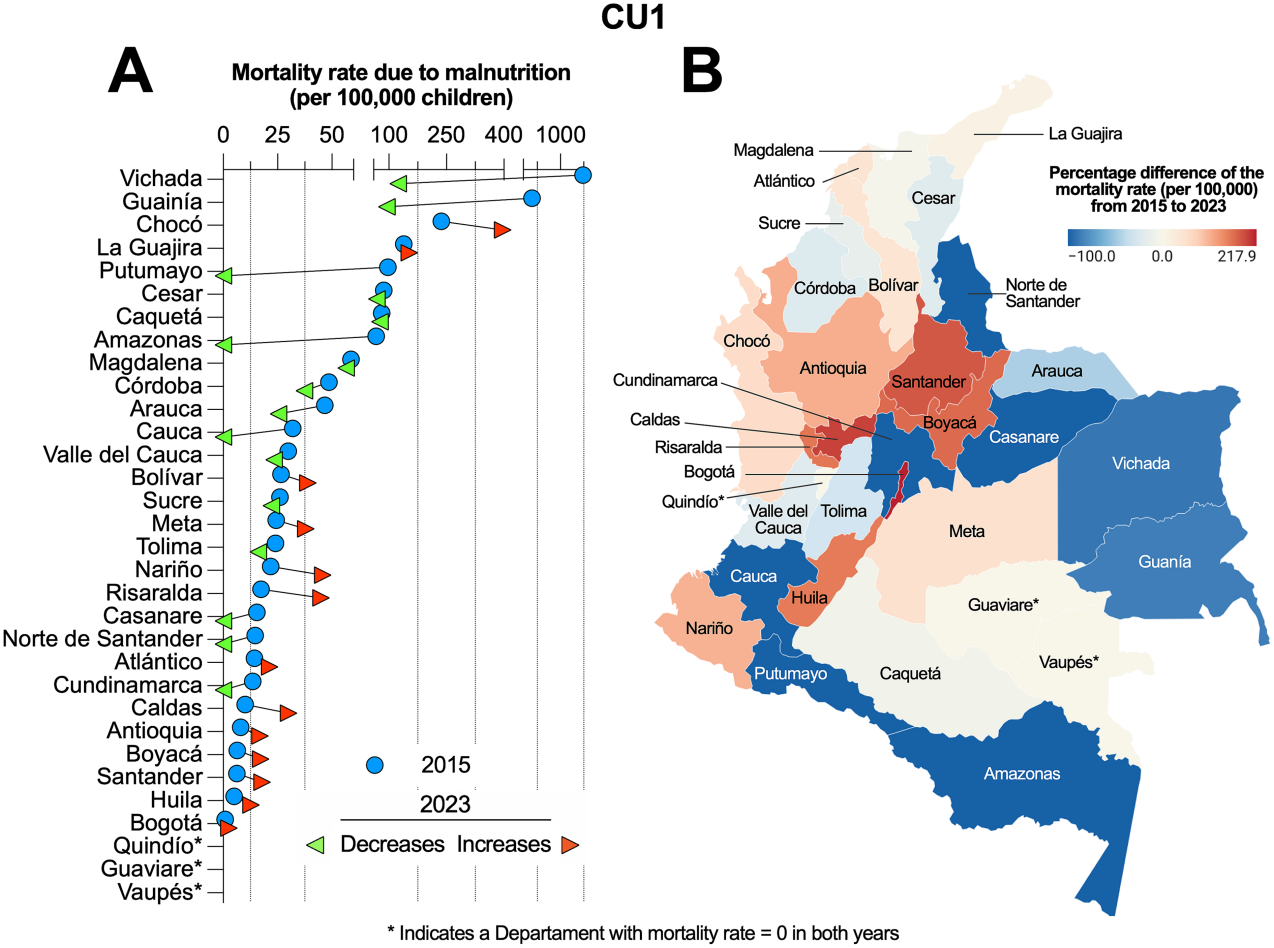
Change in the mortality rate due to malnutrition in CU1 by department in Colombia between 2015 and 2023. **(A)** Change in the mortality rate (per 100,000 children) due to malnutrition in CU1 by department from 2015 to 2023 and **(B)** spatial distribution of the percentage difference of the mortality rate from 2015 to 2023.

Figure 4 illustrates the change in the mortality rate due to malnutrition (per 100,000 children) in CU1 by department between 2015 and 2023. A total of 13 departments exhibited an increase in their rate, while other 16 exhibited a decline, though the extent of these declines varied (Figure 4A). A notable declined in mortality rates was observed in several departments, including Vichada, Guainía, Putumayo, Amazonas, and Cauca. However, in Vichada (1,243.1 to 124.1 per 100,000) and Guainía (694.4 to 94.4 per 100,000), the mortality due to malnutrition in CU1 remained among the highest in the country in 2023. At the conclusion of the period analyzed, substantial increases were documented in Chocó, where the rate rose from 237.0 to 396.5 deaths per 100,000 children, and in La Guajira, where the rate increased from 138.7 to 153.4. Conversely, Caldas, Antioquia, Boyacá, Santander, Huila, and Bogotá were the least affected departments in 2015, showing a mortality rate that ranged from 0.8 to 10.1 deaths per 100,000 children. The spatial distribution of the percentage difference in mortality rates from 2015 to 2023 exhibited a contrasting pattern of territorial changes (Figure 4B). A decline approaching 100% in mortality rates was observed in the departments of Vichada, Guainía, Putumayo, and Amazonas during the period. It is noteworthy that while certain departments, including Bogotá, Huila, Santander, and Boyacá, exhibited elevated percentage differences during the period, their mortality rates due to malnutrition in CU1 were among the lowest nationwide.

### 3.3 Analysis of the trend in mortality due to malnutrition in children under 5 at the departmental level by age group from 2015 to 2023

**Figure 5:**
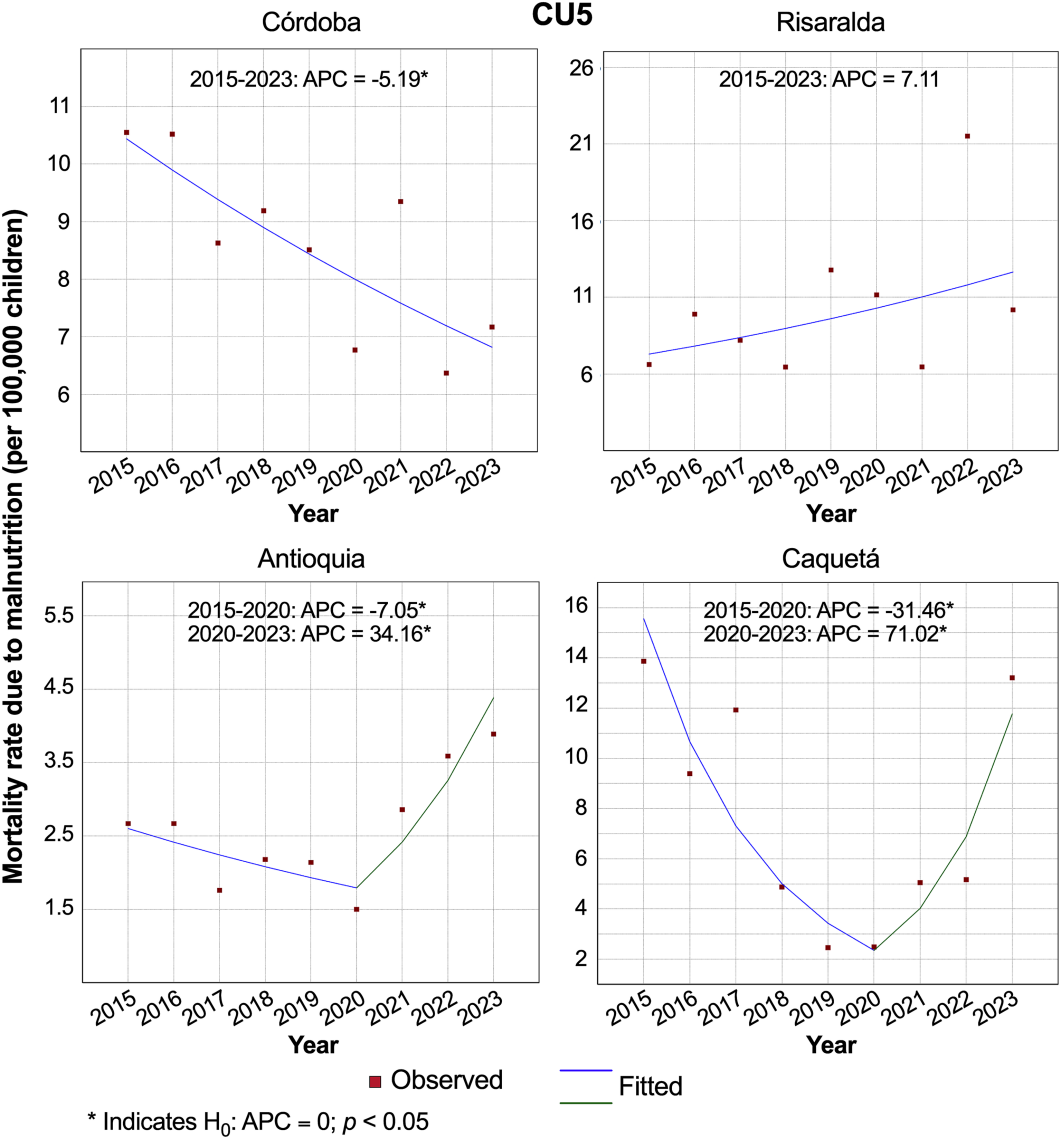
Representative results of Joinpoint trend analysis of the mortality rate due to malnutrition in CU5 in Colombia.

**Figure 6:**
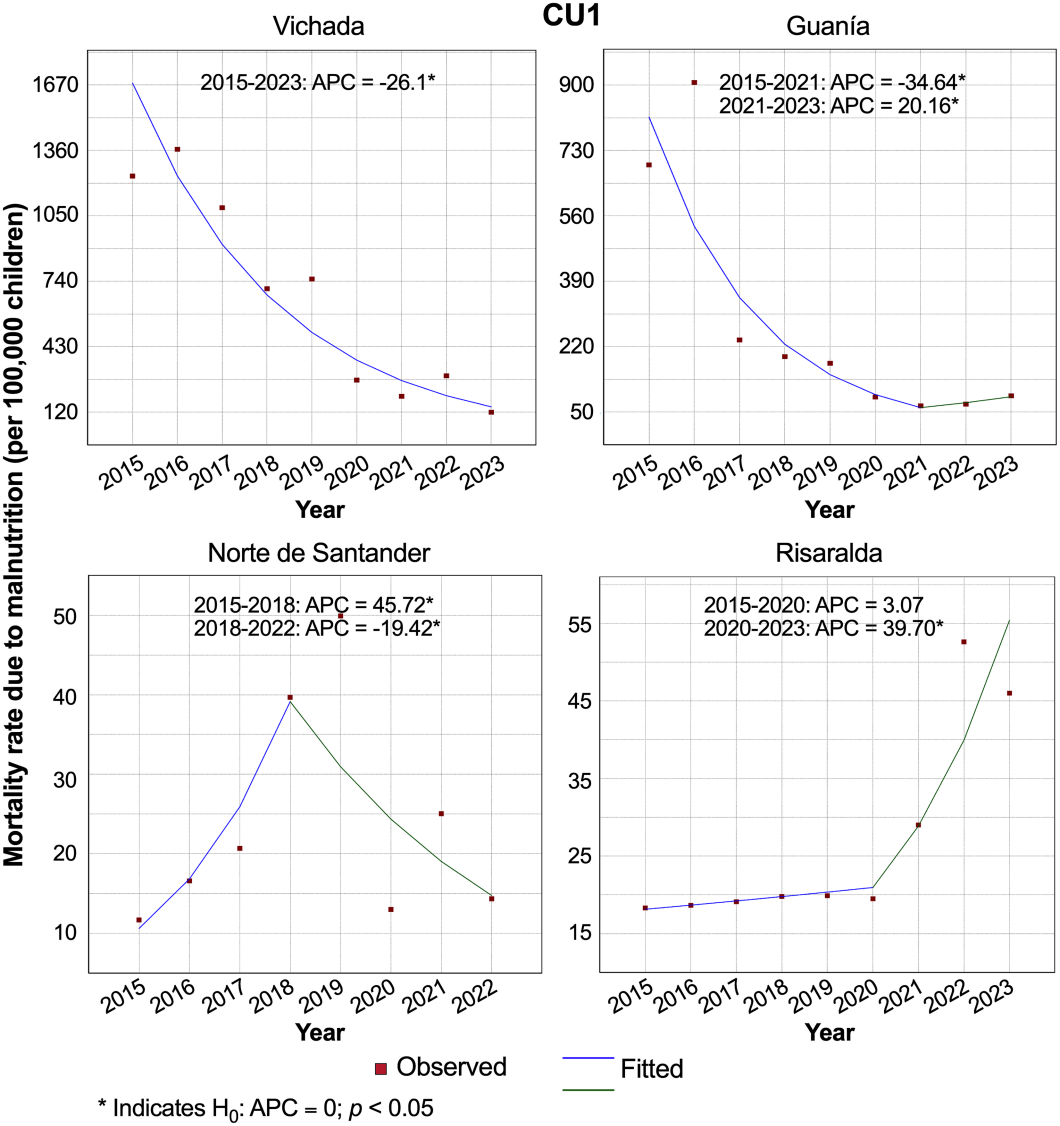
Representative results of Joinpoint trend analysis of the mortality rate due to malnutrition in CU1 year of age in Colombia.

Joinpoint trend analysis revealed a heterogeneous trend in mortality due to malnutrition in Colombian children, both between departments and within age groups (CU5 and CU1). Statistically significant changes (*p* < 0.05) were observed in at least one segment in eight departments. To illustrate the complex pattern of territorial variation, Figures 5, 6 depict representative examples of the trends found among the departments, whereas the APC and their 95% CI are presented in Table 3.

**Table 3:** Annual percentage change (APC) estimated using Joinpoint trend analysis for deaths due to malnutrition in CU5 and CU1 by department in Colombia from 2015 to 2023.

Department	CU5			CU1		
	Period	Annual percentage change (95% CI)	*p-*value	Period	Annual percentage change (95% CI)	*p*-value
Amazonas	2015–2022	11.1 (−14.4 a 45.3)	0.391	2015–2022	14.4 (−1.3 a 33.0)	0.080
Antioquia	2015–2020	−7.0 (−17.4 a − 0.3)	0.035*	2015-2020	−6.8 (−34.7 a 9.5)	0.207
	2020–2023	34.1 (15.2 a 65.2)	<0.000*	2020-2023	28.7 (−1.1 a 85.5)	0.057
Arauca	2015–2017	−32.4 (−37.1 a − 23.8)	<0.000*	2015-2023	−7.3 (−15.2 a 1.2)	0.102
	2017–2023	−1 (−4.4 a 2.1)	0.241			
Atlántico	2015–2018	40.3 (8.5 a 122.5)	0.011*	2015-2023	4.5 (−8.3 a 19.6)	0.495
	2018–2023	−11.3 (−39.1 a 1.4)	0.073			
Bogotá	2015–2023	−0.7 (−31.3 a 41.7)	0.965	2015–2023	15.3 (−4.8 a 38.3)	0.148
Bolívar	2015–2023	1.3 (−9.7 a 13.6)	0.760	2015–2023	5.1 (−4.1 a 14.8)	0.244
Boyacá	2015–2023	4.9 (−15.5 a 29.9)	0.641	2015–2023	8.9 (−0.6 a 18.4)	0.067
Caldas	2015–2023	6.6 (−7.0 a 22.4)	0.334	2015–2023	12.1 (3.9 a 21.1)	0.001*
Caquetá	2015-2020	−31.4 (−54.2 a − 18.4)	<0.000*	2015-2021	−20.5 (−58.5 a − 0.9)	0.054
	2020–2023	71.0 (15.0 a 202.2)	0.001*	2021-2023	145.0 (0.05 a 365.8)	0.049*
Casanare	2015-2022	14.2 (−1.7 a 32.7)	0.079	2015–2022	10.0 (−16.5 a 50.7)	0.456
Cauca	2015–2022	−10.5 (−24.1 a 6.27)	0.192	2015–2019	−16.6 (−41.7 a − 0.3)	0.046*
				2019-2022	37.4 (6.0 a 107.3)	0.013*
Cesar	2015-2023	−5.6 (−13.2 a 2.8)	0.171	2015–2020	−13.0 (−36.5 a 11.4)	0.006
				2020–2023	12.7 (−13.2 a 55.3)	0.239
Chocó	2015–2023	10.6 (−0.9 a 22.7)	0.051	2015–2023	8.5 (−6.2 a 26.1)	0.289
Córdoba	2015–2023	−5.2 (−8.2 a − 1.9)	0.001*	2015-2023	−4.42 (−8.3 a − 0.3)	0.034*
Cundinamarca	2015-2023	−4.3 (−20.8 a 16.7)	0.665	2015–2023	−6.5 (−30.5 a 24.9)	0.628
Guainía	2015–2023	−18.2 (−40.1 a 11.1)	0.170	2015–2021	−34.6 (−54.1 a − 26.2)	0.005*
				2021-2023	20.1 (−25.0 a 76.4)	0.600
Guaviare		ND			ND	
Huila	2015–2023	−9.7 (−18.9 a 0.5)	0.064	2015–2023	6.3 (−4.9 a 18.9)	0.286
La Guajira	2015–2023	1.9 (−8.8 a 13.7)	0.686	2015–2023	0.2 (−14.5 a 17.4)	0.984
Magdalena	2015–2023	1.9 (−2.8 a 7.2)	0.415	2015–2023	2.7 (−1.1 a 6.9)	0.173
Meta	2015–2020	−7.4 (−42.2 a 40.3)	0.246	2015–2023	1.8 (−6.8 a 11.4)	0.731
	2020–2023	31.4 (−13.9 a 111.6)	0.107			
Nariño	2015–2023	−1.7 (−22.3 a 23.9)	0.828	2015–2023	3.9 (−12.6 a 23.6)	0.628
Norte de Santander	2015–2019	25.7 (−8.7 a 200.1)	0.133	2015–2018	45.7 (11.6 a 142.7)	0.005*
	2019-2023	−33.6 (−72.5 a − 9.6)	0.035*	2018-2023	−19.4 (−48.0 a − 4.1)	0.013*
Putumayo	2015-2022	−14.3 (−40.0 a 23.0)	0.386	2015–2022	−15.6 (−32.5 a 5.7)	0.139
Quindío		ND			ND	
Risaralda	2015–2023	7.1 (−8.1 a 24.7)	0.333	2015–2020	3.01 (−8.5 a 9.4)	0.505
				2020–2023	39.7 (22.5 a 74.3)	<0.000*
Santander	2015-2023	2.5 (−6.5 a 12.4)	0.510	2015–2020	−14.9 (−65.1 a 92.8)	0.218
				2020–2023	65.1 (−29.8 a 302.1)	0.119
Sucre	2015–2023	−4.6 (−20.4 a 13.8)	0.477	2015–2023	−3.7 (−12.6 a 6.5)	0.438
Tolima	2015–2023	−5.3 (−25.1 a 20.2)	0.652	2015–2023	−3.2 (−17.4 a 14.0)	0.707
Valle del Cauca	2015–2017	−45.8 (−65.4 a − 5.2)	0.003*	2015-2017	−51.9 (−72.8 a 8.5)	0.091
	2017–2023	13.3 (−1.7 a 77.8)	0.069	2017–2023	19.9 (−12.0 a 135.8)	0.099
Vaupés	2015–2023	−6.1 (−17.3 a 6.4)	0.306		ND	
Vichada	2015–2023	−14.9 (−22.6 a 6.7)	0.000*	2015–2023	−26.1 (−39.4 a 9.5)	0.005*

*Indicates that the APC differs significantly from zero at *p* < 0.05; ND, not determined.

Regarding the mortality rates of CU5 (Figure 5), Antioquia and Caquetá exhibited divergent patterns characterized by an initial declined followed by a subsequent upward trend. Specifically, Antioquia experienced a decrease in the APC of −7.05% from 2015 to 2020, followed by a 34.16% increase between 2020 and 2023, while Caquetá had a − 31.46% APC decrease from 2015 to 2020, subsequently increasing by 71.02% from 2020 to 2023. In sharp contrast, Córdoba showed a sustained annual decrease of −5.19% throughout the period, while the mortality rate in Risaralda exhibited and increasing trend with an APC of 7.11%. A contrasting pattern was observed in the trends of the mortality rate due to malnutrition in CU1 (Figure 6). The department of Norte de Santander showed a marked upward trajectory, with a 45.72% surge during the 2015–2018 period. This was followed by a subsequent decline of −19.42% from 2018 to 2023. On the opposite, Guanía exhibited a substantial APC reduction of −34.64% from 2015 to 2021, that was followed by a 20.16% increase between 2021 and 2023. A notable upward trend was observed in Risaralda (39.7% in 2020–2023) while in contrast, Vichada exhibited a significant APC decline of −26.1% in the mortality rate of CU1.

Overall, most departments did not show significant trends (APC = 0, *p* > 0.05) in the mortality rates due to malnutrition in CU5 and CU1 (Table 3). However, significant changes in the trajectories were exhibited by Antioquia, Arauca, Atlántico, Caldas, Caquetá, Cauca, Córdoba, Guainía, Norte de Santander, Risaralda, Valle del Causa, and Vichada, which were characterized mainly by sustained or segmented decreases. However, in recent periods, certain departments, including Caquetá, Risaralda, and Antioquia, have shown concerning increases, particularly among CU1. This subnational variability may be indicative of persistent inequalities in the social determinants of malnutrition, suggesting the necessity for targeted and enhanced interventions in regions exhibiting upward or heterogeneous trends.

## 4 Discussion

The findings of this study demonstrated that deaths due to malnutrition in CU5 between 2015 and 2023 in Colombia exhibited a heterogeneous behavior in both counts and mortality rates. During the period analyzed, a downward trend in all-cause mortality was observed in CU5, accompanied by an increase in the relative weight of malnutrition as the underlying cause of death. Additionally, there were notable territorial, age, and sex inequalities. The results indicated that deaths due to malnutrition accounted for 3.58% of all deaths in CU5 between 2015 and 2023, representing an increase from 3.3 to 4.0% over the analyzed period. This observation persisted despite the documented decline in all-cause mortality within this specific age group. This trend suggests that malnutrition may play a disproportionately greater role as a cause of death among this vulnerable group. While the underlying cause may be related to the persistent and growing roles of malnutrition as a primary cause of death in early childhood in certain departments, the interpretation of this trend should be made with caution. The observed increase in the relative contribution may be due both to a real change in the risk of dying from malnutrition and more rapid declines in other causes, such as preventable infections. Public health interventions that have contributed to reducing overall child mortality, particularly from preventable causes such as acute respiratory infections and infectious diarrhea, may inadvertently highlight malnutrition as a prominent underlying cause. This phenomenon may be attributed to the accelerated decline in mortality due to infectious causes, achieved through the expansion of vaccination coverage, improvements in sanitation, and increased access to antibiotics. Consequently, malnutrition emerges as the underlying cause of death, superseding previous assumptions that attributed mortality to secondary infectious complications.

Compared to other causes of death in Colombia in 2022 among CU5, such as acute respiratory infection and diarrheal diseases with 10.7 and 2.2 deaths per 100,000, malnutrition recorded a rate of 9.7 deaths per 100,000 children (25). Although this figure is slightly lower than that for respiratory infections, its real impact is greater if malnutrition is considered as a secondary cause. Previous studies have shown that between 39.5 and 45.0% of deaths from these infectious diseases included malnutrition as a predisposing factor, thus exacerbating the influence of malnutrition on mortality among CU5 given its role as risk amplifier in vulnerable contexts (26). This syndemic pattern, in which malnutrition interacts with infections and social determinants, has been documented in Antioquia during 2021. Uribe and Ramírez (13) reported a total 43 deaths in CU5, attributable to acute respiratory infections, malnutrition, and diarrheal diseases, identifying maternal mental health and the quality of healthcare as critical factors that exacerbate child mortality.

Spatial analysis revealed differences between departments, with Chocó, La Guajira, Vichada, Guainía, and other peripheral territories consistently ranking among the highest in mortality from malnutrition in CU5. This pattern, which shows a peripheral belt of high mortality in territories far from the center of the country, is consistent with previous studies documenting high rates of mortality in this age group from preventable causes, with departmental differences related to unmet basic needs, poverty, rurality, illiteracy among women, and births to adolescent mothers, coinciding in three of the four departments with the highest preventable mortality and worst living conditions (20). In Colombia, multidimensional poverty, structural inequities, and child health challenges manifest most severely in peripheral territories distant from the country’s center. These challenges are driven by interconnected geographic, socioeconomic, and institutional factors. The predominant challenges experienced by the Amazonian, Pacific, and border departments are systemic in nature. These challenges include geographic isolation, which restricts timely access to health services, education, and labor markets; high rates of economic informality; and reliance on primary economies vulnerable to climate shocks and public security disruptions. These challenges are further exacerbated by persistent armed conflict and forced migration. The observed spatial distribution of vulnerability is indicative of longstanding political-economic centralism, a system that concentrates investments in central urban areas and perpetuates inequity gradients where chronic malnutrition and preventable mortality endure as markers of territorial exclusion. This underscores the necessity of decentralized, intersectoral approaches as a means of achieving effective mitigation.

Our results showed that, although national all-cause mortality rates among CU5 are declining, death due to malnutrition tend to increase, with both counts and rates differing markedly across departments. Such results reinforce the existence of a territorial gradient of inequality related to social determinants of health. Household-level evidence from rural Colombia indicates that food insecurity is a critical mechanism linking territorial marginalization to malnutrition outcomes. In this context, a previous study found that food insecurity, dietary transitions, and limited access to nutritious foods are the main drivers of the coexistence of child stunting and adult obesity, with these dynamics likely to be concentrated in peripheral departments where the highest mortality rates due to malnutrition occur (10). It is important to note that in some departments with small populations and low absolute numbers of deaths from malnutrition, the rates showed very marked variations. These fluctuations may reflect, in part, the influence of small denominators and the inherent instability of estimates based on few events, without altering the overall pattern of geographical inequalities identified in the country.

From an international perspective, the subnational heterogeneity observed in Colombia resembles the patterns described in Brazil and Iran, where mortality from malnutrition in CU5 has declined, but disparities between northern and southern regions or between provinces persist. Multinational studies have documented that malnutrition in CU5 shows patterns of geographical concentration in sub-Saharan Africa, South Asia, and certain territories in Latin America, associated with structural poverty, low maternal education, deficiencies in basic services, and fragmented health systems (25, 27). In low- and middle-income settings, cash transfer programs, food subsidies, and health insurance expansion have been found to be associated with significant reductions in mortality among CU5, stunting and wasting, and emaciation, with effects during times of economic recession and systemic shocks.

An important finding of this study is that CU1 consistently accounted for the highest proportion of deaths from malnutrition, representing between 57.8 and 70.2% of the deaths analyzed, which justified a specific subanalysis of this age group. This is consistent with the age distribution of infant mortality described at the global level, where the risk of death is highest in the first months of life and the decline in mortality in C1-4 is usually faster than in CU1 (28). Furthermore, it is important to note that the rates for CU1 were calculated using the number of live births as the denominator, while the rates for CU5 used population projections for this age group. Therefore, the differences observed between these two age groups reflect relative patterns, and comparisons should be interpreted with caution and within the context of their respective denominators. Additionally, it has been documented that malnutrition in all its forms is a significant risk factor during the first year of life, closely associated with low birth weight, suboptimal feeding practices, and a high burden of infections (29, 30). Furthermore, for this study, this is the only age group analyzed where death from malnutrition shows a sustained and increasing percentage change throughout the period. This behavior contrasts with that observed in C1-4, in which the event behaved heterogeneously, showing temporary decreases followed by subsequent increases, reinforcing the idea that vulnerability to malnutrition is more intense and persistent during the first year of life. This shows the importance of death due to malnutrition in CU1, reinforcing the need to prioritize interventions specifically targeting this age group, including the prevention of low birth weight, strengthening breastfeeding practices and adequate complementary feeding, as well as the timely management of infections, as part of public health strategies aimed at reducing malnutrition and preventable mortality in CU5.

Although routine surveillance of deaths from malnutrition in CU5 years of age is currently carried out, as studied in this article, this manuscript provides differential and complementary elements to the routine information derived from surveillance. It performs a detailed analysis of semiannual or annual trends and statistical breaks in mortality from malnutrition, identifying time segments with significant changes at the departmental level, which reveal a research gap on the effect of social determinants of health on the occurrence of this event. In terms of strengths, this study provides a detailed view of the impact of malnutrition on mortality in CU5 at the geographic and temporal levels, which can inform public policies aimed at reducing these gaps. However, a major limitation is the possibility of underreporting (19), especially during the pandemic years and with territorial differences, with lower quality data recording in highly rural areas, which could affect the accuracy of the reported mortality rates (31).

To examine the interdependent nature of social determinants of health (SDOH) and mortality due to malnutrition in CU5 in Colombia, we conducted a canonical correlation analysis (CCA). This multivariate statistical analysis identifies linear combinations of variables that maximize the shared variance between two sets of indicators Xs and Ys by constructing a single canonical variable V and W and maximizing the correlation between the first pair of canonical variates denoted as V_1_ and W_1_ (32). Although the description of the methodological approach and the steps taken to construct the final model is available in the Supplementary material, we present the main findings here in the discussion. This facilitated the integration and interpretation of how social determinants may interact in conjunction and how their influence on deaths due to malnutrition has changed over time in Colombia. To account for this, we constructed two models, one for the initial period that comprised the average national indicators from 2015 to 2019, and other for the final period that included the average from 2020 to 2023.

**Figure 7:**
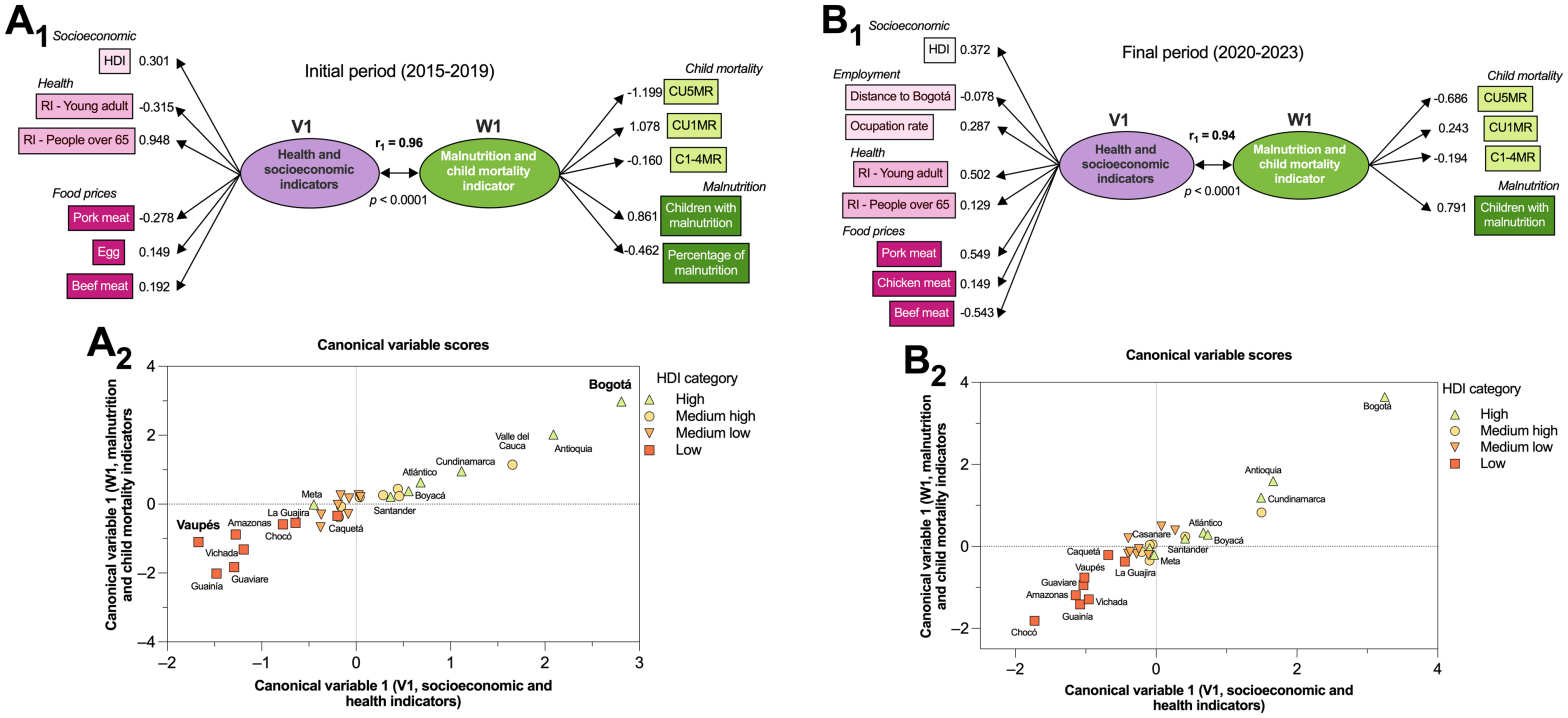
Results of canonical correlation analysis examining the association between socioeconomic indicators and child mortality and malnutrition in Colombia. Conceptual model of the association between the indicators of health and socioeconomic and malnutrition and child mortality in CU5 in Colombia **(A**_**1**_**,B**_**1**_**)** and distribution of canonical scores by department according to HDI category **(A**_**2**_**,B**_**2**_**)** for the initial (2015 to 2019) and final (2020–2023) periods. In **(A**_**2**_**)**, note the separation of extreme categories: high HDI and low HDI, with representative departments indicated.

According to the conceptual model depicted in Figure 7A_1_ for the initial period (2015–2019), CCA revealed a strong, multidimensional association between the SDOH and mortality due to malnutrition among CU5 in Colombia, although the structure of this association differed with respect to the final period examined (2020–2023, Figure 7B_1_). The results demonstrate that the SDOH are not merely a theoretical backdrop but are empirically measurable drivers of child malnutrition mortality, and their influence changed significantly between the two periods examined. In the initial period, the canonical function explained 76.7% of the shared variance, with a correlation of 0.960 (*p* < 0.0001), such linear function was predominantly defined by mortality from respiratory disease among people over 65 (standardized coefficient of 0.948) and the human development index (HDI, 0.301), with food prices bearing a comparatively lesser influence. On the dependent side, the number of children with malnutrition (0.862) and the mortality rate in CU1 (1.078) were the primary contributors, indicating that the canonical function captured both the morbidity burden and the most vulnerable age group within the first year of life. In the subsequent period (Figure 7B_1_), the explained variance increased to 83.8%, with a correlation of 0.94 (*p* < 0.0001), with food prices (especially pork and beef) emerging as the primary drivers, along with respiratory disease in young adults (0.502) and the HDI (0.372). Regarding the dependent set, the number of children with malnutrition (0.791) and the mortality rate in CU5 (−0.686) carried the greatest weight, suggesting that the association shifted toward capturing the overall mortality burden across the entire under 5 age group rather than concentrating exclusively on infants. This shift suggests that, although HDI and the burden of disease in the adult population persist as a structural pillar of the association (because older people take care of children), in the most recent period economic factors such as food prices and, to a lesser extent, employment and distance from urban centers, have emerged as relevant components of territorial vulnerability. Taken together, these findings indicate that SDOH do not operate in a static manner, instead their influence on mortality from child malnutrition is reconfigured according to prevailing economic and social conditions, reinforcing the need for intersectoral strategies adapted to each territorial context.

As depicted in Figure 7A_2_, the distribution of canonical scores by department revealed marked territorial differentiation that persisted across both periods, although with changes in the relative position of some regions. The modeling approach allows us to see that departments are not defined by isolated risk factors but by distinct profiles of advantages and disadvantages that shape child survival. To illustrate the calculation of canonical scores, we examine three departments from the initial period. Bogotá (high HDI) showed strongly positive scores (V1 = 2.89, W1 = 2.98), indicating that favorable social determinants predicted better nutritional outcomes. Vaupés (low HDI) showed markedly negative scores (V1 = −1.66, W1 = −1.10), reflecting concentrated disadvantages and worse child malnutrition outcomes. These scores are linear combinations of the original variables weighted by their canonical coefficients, demonstrating that the canonical function captures each department’s multidimensional profile rather than isolated risk factors. Accordingly, departments with a high HDI, such as Bogotá, Antioquia, and Cundinamarca, exhibited high positive scores for both social determinants and nutritional outcomes, indicating a consistent association between better structural conditions and lower mortality rates from malnutrition. Conversely, departments such as Vaupés, Vichada, and Guainía exhibited negative scores, reflecting the concentration of socioeconomic disadvantages and the suboptimal child health outcomes. Interestingly, during the initial period, the disparity between departments followed a nearly linear trajectory according to the HDI. However, in the subsequent period, more heterogeneous patterns emerged (Figure 7B_2_). For instance, some departments on the Amazonian and Pacific peripheries exhibited increased negative scores, while others, such as Casanare and Quindío, showed scores close to zero. This territorial reconfiguration suggests that the effects of recent economic and social transformations were not uniform across the country; rather, they exacerbated preexisting inequalities in some regions and altered the relationship between determinants and outcomes in others. Taken together, these findings underscore the importance of designing territorially differentiated interventions that consider both persistent structural conditions and recent changes in the social determinants of each department.

In light of the findings, recommendations are put forward for consideration, including the implementation of comprehensive programs that address both malnutrition and the social determinants of health, with a focus on regions that are especially vulnerable. For future research, it is imperative to investigate the potential integration of nutritional interventions with existing infectious disease prevention and treatment programs. Furthermore, studies that evaluate the impact of public policies targeting social determinants of health on reducing malnutrition and mortality in CU5 could provide crucial information for public health decision-making. Furthermore, it is crucial fortifying death surveillance and registration systems to guarantee that all causes of death are systematically documented, even during periods of health emergencies.

In conclusion, this study contributes to the understanding of differences in mortality rates from malnutrition in CU5 at the national and departmental levels, highlighting the importance of policies and programs that address the underlying causes of malnutrition and its interactions with other health factors. Furthermore, it suggests the need to improve registration and surveillance systems to ensure accurate data capture, especially in crisis contexts.

### 4.1 Limitations

This study offers valuable insights into the subnational patterns of child malnutrition mortality. However, it is essential to acknowledge certain limitations. First, the availability of data varies between territorial entities and years, which may introduce spatial and temporal biases in the estimates. Second, there is likely to be underreporting of malnutrition as the underlying cause of death, both due to certification errors and the prioritization of other causes in coding. This would lead to an underestimation of the actual burden of the event. Third, in this study deaths classified under ICD-10 code P07 (disorders related to short gestation and low birth weight) accounted for 7.91% of all malnutrition-attributed mortality in CU5. Although low birth weight can be indicative of maternal-fetal nutritional deficiency, it may also result from other obstetric or neonatal conditions not directly related to malnutrition. However, given the relatively minor contribution of P07-coded deaths to the total burden during the period evaluated (213 out of 2,694 deaths, Supplementary Table 1), we did not perform a sensitivity analysis excluding these cases. Nevertheless, readers should interpret mortality estimates with the understanding that this inclusion may slightly overestimate the burden strictly attributable to nutritional causes as traditionally defined. A fourth point to have in mind is the considerable number of zeros in the rates and counts of deaths from malnutrition in certain years at the departmental level complicates the application of conventional statistical methods and diminishes the capacity to discern a robust trend analysis. Fith, the use of ICD-10 codes fails to differentiate between acute and chronic forms of malnutrition, as death certificates record clinical diagnoses rather than anthropometric indicators. While this limitation is inherent to vital statistics-based research, the use of the standardized INS case definition ensures reproducibility and comparability with national surveillance data. Finally, the temporal limitation of the series, which encompassed a period less than a decade (2015–2023). This constrain precludes the analysis of long-term trends or prior cycles of the event, thereby restricting the evaluation of structural changes in the trend. Consequently, the aforementioned limitations in the available information impedes conducting more sophisticated models such as higher-resolution spatiotemporal analysis, and thus requires interpreting the findings with caution, as ecological approximations of the phenomenon rather than definitive estimates.

## Data Availability

The original contributions presented in the study are included in the article/Supplementary material, further inquiries can be directed to the corresponding authors.

## References

[1] UNICEF, WHO, The World Bank Group. Levels and Trends in Child Malnutrition: UNICEF/WHO/World Bank Group Joint Child Malnutrition Estimates. Key Findings of the 2025 Edition. Geneva: WHO (2025).

[2] Soliman A De Sanctis V Alaaraj N Ahmed S Alyafei F Hamed N . Early and long-term consequences of nutritional stunting: from childhood to adulthood. Acta Biomed Atenei Parm. (2021) 92:e2021168. doi: 10.23750/abm.v92i1.11346 PMC7975963 33682846

[3] Olofin I McDonald CM Ezzati M Flaxman S Black RE Fawzi WW . Associations of suboptimal growth with all-cause and cause-specific mortality in children under five years: a pooled analysis of ten prospective studies. PLoS One. (2013) 8:e64636. doi: 10.1371/journal.pone.0064636, 23734210 PMC3667136

[4] McDonald CM Olofin I Flaxman S Fawzi WW Spiegelman D Caulfield LE . The effect of multiple anthropometric deficits on child mortality: meta-analysis of individual data in 10 prospective studies from developing countries. Am J Clin Nutr. (2013) 97:896–901. doi: 10.3945/ajcn.112.047639, 23426036

[5] Troeger CE Arndt MB Aalruz H Abdoun M Abdullahi A Abebe M . Quantifying the fatal and non-fatal burden of disease associated with child growth failure, 2000–2023: a systematic analysis from the global burden of disease study 2023. Lancet Child Adolesc Health. (2000) 10:22–38. doi: 10.1016/S2352-4642(25)00303-7, 41344792 PMC12674951

[6] Agudelo-Ibañez DR. Mortalidad por desnutrición infantil en menores de 5 años y sus determinantes a nivel municipal en Colombia 1998-2016. Departamento de Salud Pública, Facultad de Medicina, Universidad Nacional de Colombia. Bogotá, Colombia (2019)

[7] Abdu AN Murugan R Tilahun SW. Survival status and predictors of mortality among severely acute malnourished under-5 children admitted to stabilisation centers in selected government hospitals in Addis Ababa, Ethiopia, 2022: a retrospective cohort study. BMJ Open. (2024) 14:e083855. doi: 10.1136/bmjopen-2023-083855, 39107018 PMC11308885

[8] Rebouças P Goes E Pescarini J Ramos D Ichihara MY Sena S . Ethnoracial inequalities and child mortality in Brazil: a nationwide longitudinal study of 19 million newborn babies. Lancet Glob Health. (2022) 10:e1453–62. doi: 10.1016/S2214-109X(22)00333-3, 36113530 PMC9638038

[9] Uribe-Quintero R Álvarez-Castaño LS Caicedo-Velásquez B Ruiz-Buitrago IC. Trends in undernutrition mortality among children under five years of age and adults over 60. Biomedica. (2022) 42:41–53. doi: 10.7705/biomedica.5937, 35471169 PMC9048577

[10] Sansón-Rosas AM Bernal-Rivas J Kubow S Suarez-Molina A Melgar-Quiñonez H. Food insecurity and the double burden of malnutrition in Colombian rural households. Public Health Nutr. (2021) 24:4417–29. doi: 10.1017/S1368980021002895, 34218842 PMC10195310

[11] Lamus-Lemus F Díaz-Quijano DM Rincón-Rodríguez CJ Huertas-Moreno ML. Avances en la comprensión de la transición nutricional colombiana. Rev Gerenc Polit Salud. (2012) 11:121–33.

[12] Instituto Colombiano de Bienestar Familiar, Instituto Nacional de Salud. Encuesta Nacional de la Situación Nutricional (ENSIN 2015). Bogotá, DC: Gobierno de Colombia: Instituto Nacional de Salud (2019).

[13] Uribe JCG Ramírez VCC. Mortalidad infantil en Antioquia durante el año 2021: hacia un enfoque sindemico. Rev Cienc Cuid. (2021) 20:39–50. doi: 10.18273/saluduis.55.e:23015

[14] Forero Ballesteros LC Forero Torres AY. Tendencia temporal de la mortalidad por desnutrición en Colombia, 2005-2019. Rev Panam Salud Publica. (2023) 46:1. doi: 10.26633/RPSP.2022.4, 35299716 PMC8922941

[15] Quiroga EF. Mortalidad por desnutrición en menores de cinco años, Colombia, 2003-2007. Biomedica. (2003) 32:499–509. doi: 10.7705/biomedica.v32i4.741 23715225

[16] Bhutta ZA Das JK Rizvi A Gaffey MF Walker N Horton S . Evidence-based interventions for improvement of maternal and child nutrition: what can be done and at what cost? Lancet. (2013) 382:452–77. doi: 10.1016/s0140-6736(13)60996-4, 23746776

[17] Morgenstern H. Ecologic studies in epidemiology: concepts, principles, and methods. Annu Rev Public Health. (1995) 16:61–81. doi: 10.1146/annurev.pu.16.050195.000425, 7639884

[18] INS. Protocolo de vigilancia en salud pública: mortalidad por y asociada a desnutrición en menores de cinco años. Bogotá, DC: Gobierno de Colombia: Instituto Nacional de Salud (2015).

[19] Rojas-Botero ML Borrero-Ramlrez YE Fernández-Niño JA. Evaluación de la calidad de las estadísticas vitales de niños menores de cinco años. Colombia, 2000-2018. Rev Univ Ind Santander Salud. (2000) 55:1–11. doi: 10.18273/saluduis.55.e:23015

[20] Rojas-Botero ML Fernández-Niño JA Borrero-Ramírez YE. Inequality trajectories in avoidable under-5 mortality in Colombia: a 23-year analysis of inequities (2000-2022). SSM Popul Health. (2025) 30:101782. doi: 10.1016/j.ssmph.2025.101782, 40212737 PMC11982479

[21] DANE. Estadísticas vitales: nacimientos y defunciones. Bogotá, DC: Gobierno de Colombia. Departamento Administrativo Nacional de Estadística (2025). Available online at: http://www.dane.gov.co/index.php/estadisticas-por-tema/demografia-y-poblacion/nacimientos-y-defunciones. (accessed [December 19, 2025]).

[22] DANE. Proyecciones de población basadas en el Censo Nacional de Población y Vivienda Bogotá, DC: Gobierno de Colombia Departamento Administrativo Nacional de Estadística (2025). Available online at: https://www.dane.gov.co/index.php/estadisticas-por-tema/demografia-y-poblacion/proyecciones-de-poblacion (accessed November 02, 2025).

[23] Beltran-Ontiveros SA Fernandez-Galindo MA Moreno-Ortiz JM Contreras-Gutierrez JA Madueña-Molina J Arambula-Meraz E . Incidence, mortality, and trends of prostate Cancer in Mexico from 2000 to 2019: results from the global burden of disease study 2019. Cancer. (2022) 14:3184. doi: 10.3390/cancers14133184, 35804962 PMC9265044

[24] DANE. Estadísticas Vitales (EEVV). Bogotá, DC: Gobierno de colombia. Departamento Administrativo Nacional de Estadística (2023).

[25] Amir-Ud-Din R Fawad S Naz L Zafar S Kumar R Pongpanich S. Nutritional inequalities among under-five children: a geospatial analysis of hotspots and cold spots in 73 low- and middle-income countries. Int J Equity Health. (2022) 21:135. doi: 10.1186/s12939-022-01733-1, 36104780 PMC9476341

[26] Wang Y Han R Ding X Chen J Feng W Wang C . A 32-year trend analysis of lower respiratory infections in children under 5: insights from the global burden of disease study 2021. Front Public Health. (2025) 13:1483179. doi: 10.3389/fpubh.2025.1483179, 39911225 PMC11794078

[27] Flores-Quispe MDP Restrepo-Méndez MC Maia MFS Ferreira LZ Wehrmeister FC. Trends in socioeconomic inequalities in stunting prevalence in Latin America and the Caribbean countries: differences between quintiles and deciles. Int J Equity Health. (2019) 18:156. doi: 10.1186/s12939-019-1046-7, 31615530 PMC6794733

[28] Karlsson O Kim R Hasman A Subramanian S. Age distribution of all-cause mortality among children younger than 5 years in low-and middle-income countries. JAMA Netw Open. (2022) 5:e2212692. doi: 10.1001/jamanetworkopen.2022.12692, 35587349 PMC9121187

[29] Kefale B Jancey J Gebremedhin AT Nyadanu SD Belay DG Pereira G . Risk factors of under-five and infant mortality: an umbrella review of systematic reviews and meta-analyses. J Glob Health. (2024) 14:04260. doi: 10.7189/jogh.14.04260, 39611446 PMC11605776

[30] Li Y Xu J Li Z Ren M Jiang S. Burden of common infectious diseases in children with growth failure from 1990 to 2021: analysis of the global burden of disease study. Front Pediatr. (2025) 13:1648964. doi: 10.3389/fped.2025.1648964, 41278030 PMC12634535

[31] Chaparro-Narváez P Manrique Sanchez JA Berrio-Parra L Urrego Ricaurte DC Torres-Rojas LJ Orjuela Cantor NP . Accuracy of information on the underlying cause of death: an analysis in Colombia during the COVID-19 pandemic in 2021. PLoS One. (2025) 20:e0320466. doi: 10.1371/journal.pone.0320466, 40393038 PMC12092014

[32] Ayala-Azcárraga C Diaz D Zambrano L. Characteristics of urban parks and their relation to user well-being. Landsc Urban Plan. (2019) 189:27–35. doi: 10.1016/j.landurbplan.2019.04.005

