# Supplementary material for: Deaths due to malnutrition in children under five in Colombia, 2015–2023: a spatiotemporal analysis
Source: Front Public Health. 2026 Jun 22;14:1816399. doi: 10.3389/fpubh.2026.1816399 (PMC13333647; doi:10.3389/fpubh.2026.1816399)
Supplement: Supplementary file 1 [file Table_1.DOCX]

Supplementary Material

**Supplementary Table 1.** Deaths due to ICD-10 code P07 (short gestation/low birth weight).

| **Year** | **Deaths due to malnutrition in children under 5 years of age** | **Deaths attributable to ICD-10 code P07** | **%** |
| --- | --- | --- | --- |
| 2015 | 286 | 24 | 8.392 |
| 2016 | 348 | 12 | 3.448 |
| 2017 | 241 | 10 | 4.149 |
| 2018 | 351 | 22 | 6.268 |
| 2019 | 301 | 28 | 9.302 |
| 2020 | 242 | 28 | 11.570 |
| 2021 | 277 | 22 | 7.942 |
| 2022 | 365 | 38 | 10.411 |
| 2023 | 283 | 29 | 10.247 |
| **Total** | **2,694** | **213** | **7.906** |

**Supplementary Analysis**

Statistical Analysis

Canonical correlation analysis (CCA) was used to determine the association between a set of indicators related to SDOH and mortality due to malnutrition among CU5 of age in 2015 and 2023, with the aim of comparing the influence of these determinants on outcomes related to morbidity and mortality from malnutrition across two analysis periods. The objective of CCA is to find a linear relationship between sets of multiple dependent and independent variables, maximizing the shared variance (Afifi et al., 2019), which makes it suitable for ecological-level research involving aggregated national indicators. For this analysis, a weighted average of the variables in the first set was calculated and correlated with a weighted average of the variables in the second set. The weighted averages of the original variables are called canonical variables. CCA finds the linear combination of independent variables (X1, X2, etc.) and the linear combination of dependent variables (Y1, Y2, etc.) that maximizes the correlation. Each canonical function is constructed based on the correlation between two canonical variables, one from the dependent variables and the other from the independent variables.

We constructed two models, one for the initial period that comprised the average national indicators from 2015 to 2019, and other for the final period that included the average from 2020 to 2023.

The dependent and independent variables in this study were defined as follows:

Dependent variables:

- Mortality rate due to malnutrition in children under 5 years of age: Mortality rates due to malnutrition were calculated for the following three groups: 1) children under five years of age (0–4 years, per 100,000 children under five), 2) children under 1 year of age (0–1 years, per 100,000 live births), and 3) children aged 1–4 years (1–4 years, per 100,000 children aged 1–4 years). To calculate each of the rates, the number of deaths due to malnutrition in each age group was used as the numerator, while the population projections for each group were used as the denominator, and the resulting ratio was multiplied by 100,000. These rates were calculated annually for each department.

- Number of cases of malnutrition in children under 5 years of age: A case of acute malnutrition in children under 5 years of age is defined when the z-score of the weight-for-height or length indicator is below -2SD and/or the child exhibits the phenotypes of severe acute malnutrition (marasmus, kwashiorkor, or marasmic kwashiorkor). It is associated with recent weight loss or an inability to gain weight, of primary etiology, caused in most cases by low food intake and/or the presence of infectious diseases. Excluded from this diagnosis are children with malnutrition of secondary etiology, with a history of low birth weight and whose weight loss cannot be attributed to acute malnutrition, and children with a history of prematurity who cannot

- Prevalence of malnutrition: percentage of children under 5 years of age who are malnourished out of the total population of children under 5 years of age for the period.

Independent variables:

- Food prices: annual average price of protein-rich foods, reported in Colombian pesos per unit of purchase, for the main food distribution center serving each department. For beef, pork, and chicken, the unit of purchase is the kilogram; for eggs, the unit is the egg. Prices are taken from the DANE’s Price Information System (SIPSA).

- Human Development Index (HDI): a measure that summarizes a population’s achievements in a single indicator using a geometric mean of three standardized indices (health, education, and income), calculated by the United Nations Development Programme (UNDP)

- Unemployment rate: the percentage of the economically active population that is unemployed but available for work and actively seeking employment during a given period.

- Number of deaths from acute respiratory infections: the number of reported deaths from respiratory diseases among individuals aged 18 and older.

The assumptions of the CCA are linearity, multicollinearity (high multiple correlation among the dependent or independent set), and multivariate normality. These assumptions were verified using exploratory diagnostics and correlation matrices. Statistical significance was set at an alpha level of 0.05. The statistical analysis was performed using SAS OnDemand for Academics software (SAS Institute Inc., Cary, NC, USA), employing standard procedures for data management, cleaning, and modeling.

**Supplementary Results of the CCA models**

Initial model (2015 to 2019)

The final canonical correlation model was constructed and analyzed, consisting solely of the six selected independent variables (canonical variable V1) and the five dependent variables (canonical variable W1). The final model yielded two statistically significant canonical functions, with the first canonical function showing a correlation of 0.960 (*p* < 0.0001), which explained 76.7% of the shared variance between the two groups of indicators. The second function, with a correlation of 0.874 (*p* = 0.0057), explained an additional 21.1%, resulting in a cumulative explained variance of 97.5% between the two. One multivariate tests corroborated its overall significance (Wilks’ Lambda = 4.98, d.f. = 30 num / 78 den; *p* < 0.0001), confirming that the association between the set of socioeconomic and health indicators and that of infant mortality and malnutrition is robust and multidimensional.

Final model (2020 to 2023)

The set of independent variables included eight indicators for the canonical variable V1, while the dependent canonical variable W1 consisted of four indicators. Only the first canonical correlation was significant (*p* < 0.05), with a value of 0.957 and 83.8% of variance explained, demonstrating that the selected indicators are not only closely associated but that the constructed model also has a high capacity to explain the intrinsic variance of the dataset. Furthermore, this association was corroborated by the results of the Wilks’ Lambda test (4.07, d.f. = 32 num / 67.9 den; *p* < 0.0001), which demonstrated the overall significance of the final model

References

AFIFI, A., MAY, S., DONATELLO, R. A. & CLARK, V. A. 2019. *Practical multivariate analysis*, Chapman and Hall/CRC.
